# SUMOylation Regulates Neutrophil Phagocytosis and Migration

**DOI:** 10.3390/ph18071070

**Published:** 2025-07-20

**Authors:** Ran Zhang, Wanying Miao, Jin Zhang, Xinyuan Yu, Lihong Dang, Ata Ur Rehman, Feng Xu, Huaxin Sheng, G. Chad Hughes, Joseph P. Mathew, Jörn Karhausen, Wei Yang

**Affiliations:** 1 Department of Anesthesiology, Duke University Medical Center, Durham, NC 27710, USA; 2 Department of Surgery, Division of Thoracic and Cardiovascular Surgery, Duke University Medical Center, Durham, NC 27710, USA; 3 Department of Anesthesiology, IRCCS Humanitas Research Hospital, 20089 Rozzano, MI, Italy; 4 Department of Neurology, Duke University Medical Center, Durham, NC 27710, USA

**Keywords:** neutrophil plasticity, TAK981, hypothermia, immune response, phagocytosis

## Abstract

**Introduction**: Accumulating evidence indicates that neutrophils undergo reprogramming of their effector functions as they migrate from the bloodstream into an inflamed tissue. Here, we examined the role of the small ubiquitin-like modifier (SUMO) conjugation in modulating neutrophil functional changes in the inflammatory microenvironment. **Methods**: Primary human and murine neutrophils were used to assess SUMOylation levels in vitro by Western blotting and results were validated in clinical samples from patients undergoing surgery involving hypothermic circulatory arrest. SUMOylation was inhibited with TAK-981, and its impact on neutrophil migration, NETosis, and phagocytosis was assessed in vitro. The in vivo effect of TAK-981 on neutrophil tissue infiltration was further evaluated using a sterile sponge assay in mice. **Results**: Our results demonstrated that neutrophil SUMOylation was induced by factors of the inflammatory microenvironment (temperature and oxidative stress) and inflammatory stimulants in vitro, and under conditions of general inflammatory activation in patients. Further, we found that blocking SUMOylation with TAK-981 in vitro blunted neutrophil migration and phagocytosis but did not affect NETosis. Notably, TAK-981 treatment reduced neutrophil accumulation in sterile sponges in mice. **Conclusions**: Our work identifies SUMOylation as a novel mechanism of neutrophil tissue reprogramming. Blocking SUMOylation may provide a therapeutic option to limit the contribution of neutrophils to inflammation-associated tissue damage.

## 1. Introduction

Neutrophils are critical effector cells in the innate host defense. At infection sites, they eliminate pathogens using multiple mechanisms and critically shape the local inflammatory environment through direct and indirect communication with resident immune cells and cells of the adaptive immune system [1]. Recent research has shown that neutrophils, once considered to be functionally homogeneous and terminally differentiated cells, can adapt their function and migration behavior to the specific inflammatory environments depending on the tissue and the intensity of inflammation [2,3]. While mechanisms like neutrophil homing via specific cytokines have been studied [4,5], it remains incompletely understood how microenvironmental conditions may reprogram neutrophil phenotypes.

In our work, we hypothesized that the environmental conditions present at sites of inflammation may modulate SUMOylation levels in transmigrating neutrophils, thereby altering their functions. SUMOylation, a post-translational modification attaching small ubiquitin-like modifier (SUMO) proteins to target proteins, regulates diverse cellular activities, including transcription factors, enzyme and receptor activity, cellular trafficking, nuclear protein assembly, chromatin remodeling, and protein–protein interactions [6]. Vertebrates express three main SUMO isoforms. SUMO1 is found mainly conjugated to high-affinity targets, whereas conjugation with the highly homologous SUMO2 and 3 isoforms (often combined as SUMO2/3) is inducible by stressors such as heat shock [7], oxidative stress [8], hypoxia [9]—hence, conditions often found at inflammation sites. The increase in SUMO2/3 conjugation represents the modification of a broad array of target proteins and causes the reprogramming of complex cellular responses with prominent consequences for several aspects of inflammation and innate immunity [6,10,11,12,13,14]. However, while neutrophils are exposed to drastic microenvironmental changes as they transmigrate from the bloodstream into inflamed tissues, the role of SUMOylation in modulating neutrophil responses has not yet been defined.

Notably, such research has been impeded by the lack of effective ways to modify SUMOylation, as genetic targeting of one SUMO isoform can lead to compensatory overexpression of the others. Recently, a group of adenosine sulfamate-derived inhibitors of SUMOylation was identified that acts by forming a SUMO-inhibitor adduct as the inhibitory species within the catalytic site of the E1 SUMOylation complex [15]. Ensuing optimization of specificity and adduct stability has yielded in TAK-981 [16], a first-in-class SUMOylation inhibitor that has demonstrated potent activity in various forms of cancer, where increased SUMOylation strongly supports abnormal cell growth [17]. TAK-981 thus constitutes a powerful tool for mechanistic studies in conditions associated with increased SUMOylation. The objective of this study was to examine if neutrophil SUMOylation was altered in response to environmental stimuli and whether resultant changes in SUMOylation levels impacted essential neutrophil responses. Using TAK-981 as an inhibitor of SUMOylation not only provides first mechanistic insights into the consequences of SUMO inhibition in neutrophils but also establishes early data on the therapeutic potential of targeting SUMOylation to modify the neutrophil phenotype in disease conditions associated with neutrophil infiltration.

## 2. Results

### 2.1. Environmental and Inflammatory Stimuli Increase Neutrophil SUMOylation In Vitro and In Vivo

**Figure 1:**
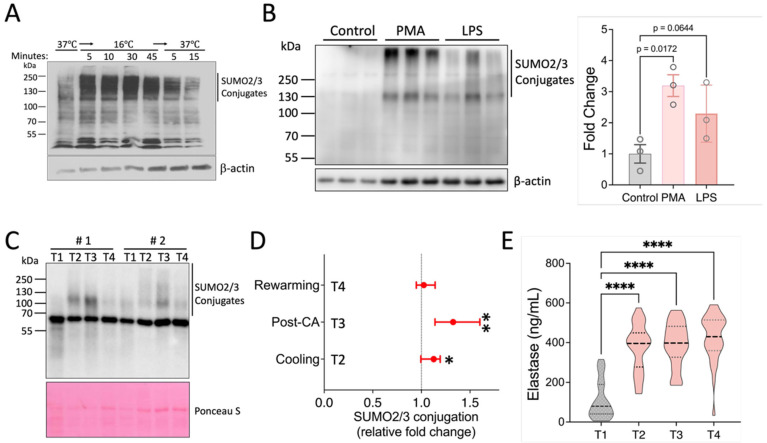
Activation of SUMO2/3 conjugation in response to various environmental and inflammatory stimuli. (**A**) Activation of SUMO2/3 conjugation in human neutrophils by hypothermia. (**B**) SUMO2/3 activation by PMA and LPS. Data are presented as mean ± SEM. (**C**–**E**) Patient samples. Samples (*n* = 49) were collected at four timepoints (T1–4) from patients undergoing cardiopulmonary bypass as described in the Methods. (**C**,**D**) Western blot analysis of SUMO2/3 conjugation in leukocytes enriched from blood samples of 49 patients (in total, 196 samples). Representative Western blot data from two patients are shown here. Fold changes were calculated relative to T1 samples for each patient and are presented as median values with 95% confidence intervals (CIs). *p*-values for each group were determined using a one-sample *t*-test. CA: circulatory arrest. (**E**) Serum elastase levels. Violin plots are shown (*n* = 31). *, *p* < 0.05; **, *p* < 0.01; ****, *p* < 0.0001.

To investigate whether SUMOylation functions as an important regulator of neutrophil behaviors, we first aimed to determine how SUMOylation levels change when neutrophils are exposed to typical stressors. Here, we had previously observed that a substantial increase in global SUMOylation is induced by hypothermia [18], and consequently exposed isolated human neutrophils from healthy volunteers to normothermia and 16 °C before examining SUMO2/3 conjugation by Western blotting. As shown in Figure 1A, SUMO2/3 conjugation increased rapidly as evidenced by the presence of high molecular smear denoting a broad range of SUMO-modified proteins after exposure to hypothermia. Importantly, this increase was reversible when cells were rewarmed. Further supporting the notion that SUMOylation is responsive to inflammatory stimuli, we exposed isolated mouse neutrophils to phorbol-12-myristate-13-acetate (PMA) and lipopolysaccharide (LPS). We found that SUMO2/3 conjugation was increased in response to these inflammatory stimulants (Figure 1B). Together, neutrophil SUMOylation is highly responsive to environmental and inflammatory stimuli.

We next sought to establish dynamic neutrophil SUMOylation in a clinical context. In cardiac surgery with cardiopulmonary bypass (CPB), a robust systemic inflammation is triggered in a predictable manner, allowing us to obtain samples at the baseline (before the initiation of surgery) and at various stages during surgery [19]. Specifically, we examined patients undergoing aortic surgery which, in addition to extracorporeal circulation, necessitates cooling of the body and a brief period of circulatory arrest for completing the aortic anastomoses. Therefore, this procedure introduces multiple inflammatory stimuli, including surgical trauma, blood contact with artificial surfaces, ischemia/reperfusion (I/R), and changes in body temperature. Western blot analysis of leukocyte samples obtained from 49 patients at four timepoints of surgery documented that compared to baseline, CPB perfusion and whole-body cooling led to an increase in the high-molecular “smear” representing SUMOylated proteins (Figure 1C,D and Figure S1). Quantification of the signal intensity indicated that SUMOylation remained significantly elevated immediately after circulatory arrest but decreased rapidly with rewarming and separation from bypass (Figure 1D). As the parent study was performed with randomized cooling regimens at the time of the circulatory arrest (deep [≤20.0 °C], vs. low-moderate [20.1–24.0 °C], and high-moderate hypothermia [24.1–28.0 °C]), we examined potential differences in SUMOylation levels in these groups. In agreement with our earlier data in a rat model [20], SUMOylation was induced with high-moderate hypothermia, but was not significantly augmented in the lower temperature groups. However, while we attempted to preserve the snapshot SUMOylation levels at the point of blood draw through the addition of inhibitors of de-SUMOylation and performance at 4 °C, the complexity of the procedure to obtain protein from patient white blood cells may also have precluded us from safely making more detailed distinctions and thus we decided to pool the data from the three treatment groups.

We next contextualized these SUMOylation patterns with markers of neutrophil activation. We found a significant increase in neutrophil elastase serum levels upon the initiation of extracorporeal circulation and cooling (374.7 ± 120 ng/mL), compared to the baseline (109.4 ± 96.4 ng/mL, *p* < 0.001), with neutrophil activation increasing further during the observation period to a maximum of 426.7 ± 120.0 ng/mL after rewarming and separation from the CPB. These findings indicate that the observed increase in SUMOylation occurred in the context of robust neutrophil activation initiated by CPB perfusion and whole-body cooling (Figure 1E).

Together, our results therefore document dramatic changes in leukocyte and neutrophil SUMOylation under conditions of cellular stress and inflammation and identify SUMOylation as a potentially important modulator of inflammatory effector functions.

### 2.2. Inhibiting the SUMOylation Response Differentially Regulates Effector Functions in Isolated Murine Neutrophils

**Figure 2:**
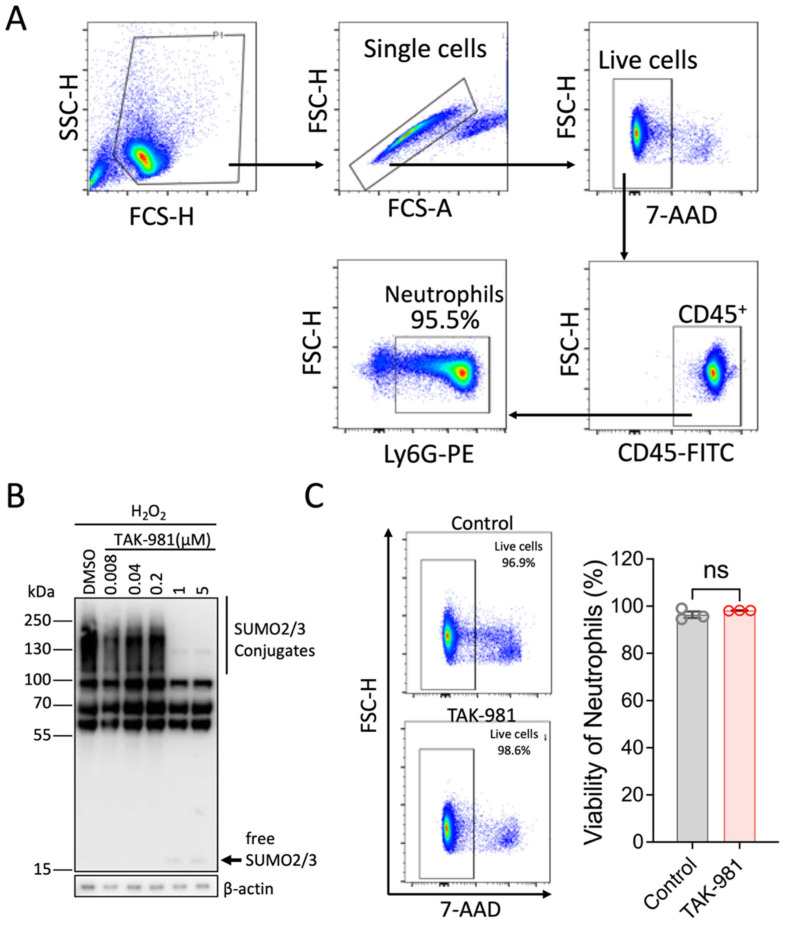
Characterization of isolated neutrophils for in vitro studies. (**A**) The purity of isolated neutrophils was analyzed by flow cytometry. (**B**) TAK-981 effectively inhibits SUMOylation in neutrophils. Hydrogen peroxide was used to increase the levels of SUMOylation. Different doses of TAK-981 were tested. (**C**) TAK-981 at 5 μM is not toxic to neutrophils. Neutrophils were incubated with TAK-981 (5 μM) for 2 h and then cell viability was analyzed using 7-AAD staining. Data are presented as mean ± SEM (*n* = 3/group); ns, not significant.

Given that all tested stimuli increased global SUMOylation levels, we next examined how neutrophil functions were affected when the SUMOylation response was blocked. To this end, we first isolated murine primary neutrophils and confirmed the high purity of the samples (Figure 2A). We then assessed the blocking efficiency of TAK-981 in neutrophils. To facilitate the detection of SUMOylation, we exposed neutrophils to oxidative stress (Figure 2B). As expected, TAK-981 effectively suppressed the SUMOylation increase induced by hydrogen peroxide at doses above 1 μM (Figure 2B). Of note, we confirmed that the dose of TAK-981 at 5 μM had no effect on cell viability (Figure 2C).

**Figure 3:**
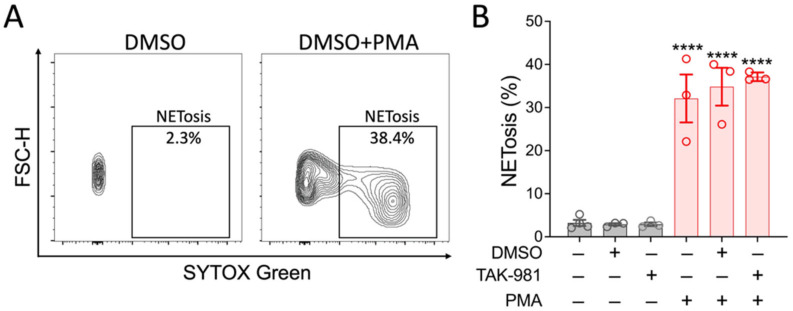
TAK-981 has no effect on NETosis activity of neutrophils. Neutrophils were pretreated with TAK-981 or DMSO (vehicle) for 1 h. Then, cells were stimulated with PMA for 2 h, followed by flow cytometry analysis. (**A**) Representative flow cytometry plots. (**B**) Percentage of NETosis positive neutrophils. Data are presented as mean ± SEM (*n* = 3/group). ****, *p* < 0.0001.

**Figure 4:**
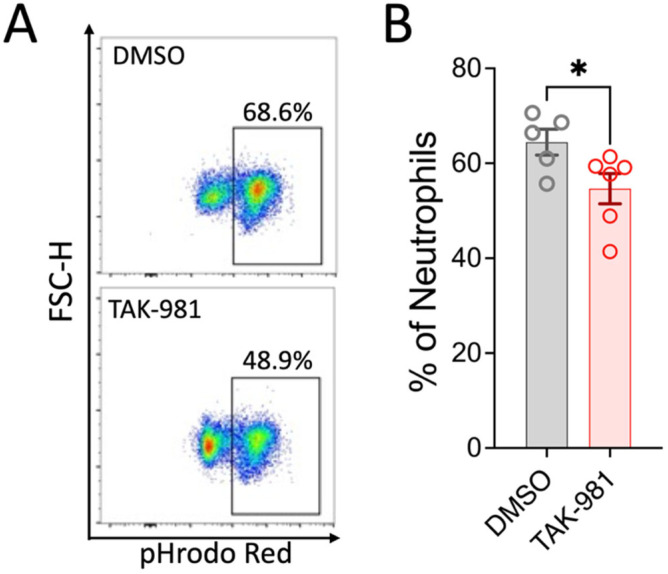
TAK-981 inhibits phagocytosis activity of neutrophils. Neutrophils were pretreated with TAK-981 or DMSO (vehicle) for 1 h. Then, cells were incubated with pHrodo Red bioparticles for another hour, followed by flow cytometry analysis. (**A**) Representative flow cytometry plots. (**B**) Percentage of pHrodo Red positive neutrophils. Data are presented as mean ± SEM (*n* = 5–6/group). *, *p* < 0.05.

**Figure 5:**
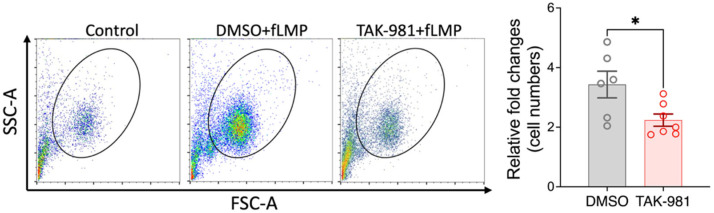
TAK-981 suppresses migration ability of neutrophils. Transwell migration assay was performed. Cells that migrated into the lower chamber were counted with flow cytometry and fold changes relative to the control group were calculated. Data are presented as mean ± SEM (*n* = 6–7/group). *, *p* < 0.05.

Activated neutrophils can release decondensed chromatin along with granular and cytosolic components to form neutrophil extracellular traps (NETs), a process called NETosis. To examine the effect of blocking SUMOylation on NETosis, we stimulated neutrophils with PMA, and the extracellular DNA of NETs was stained with SYTOX Green for flow cytometry analysis. Our results indicate that TAK-981 did not significantly alter NETosis (34.8 ± 4.4% NETosis in DMSO control vs. 37.1 ± 0.6% in TAK-981 treatment; Figure 3). Next, we analyzed phagocytic activity, a crucial function of neutrophils to eliminate pathogens and cell debris. Notably, we observed that TAK-981 treatment caused a slight but significant reduction in the phagocytic activity in neutrophils (Figure 4). Lastly, as neutrophil tissue migration is a prerequisite to the innate immune response to injury or infection, we used an in vitro transwell assay to evaluate this neutrophil function. We found that pretreatment with TAK-981 significantly reduced fMLP-driven neutrophil migration (Figure 5).

Together, these findings support that SUMOylation differentially affects certain essential neutrophil functions and identifies an important role of SUMOylation in the regulation of neutrophil phagocytosis and migration.

### 2.3. TAK-981 Inhibits Neutrophil Tissue Infiltration In Vivo

**Figure 6:**
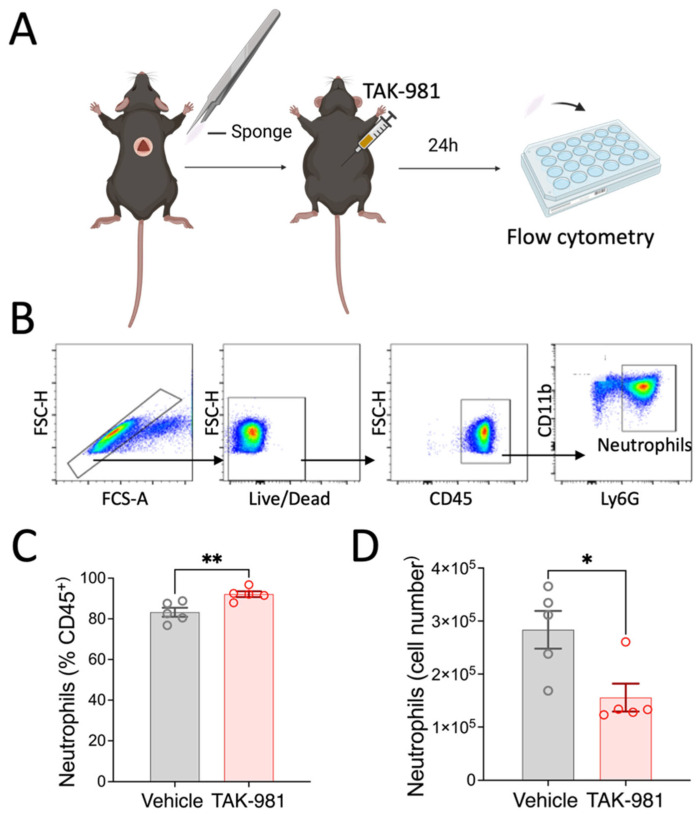
Effects of TAK-981 treatment on neutrophil migration into implanted sponge. (**A**) Experimental design. (**B**) Gating strategy. (**C**,**D**) Frequency and cell numbers of neutrophils isolated from sponges. Neutrophils were gated as CD45^+^CD11b^+^Ly6G^+^ cells. Data are presented as mean ± SEM (*n* = 5/group). *, *p* < 0.05; **, *p* < 0.05.

The first wave of the immune response to tissue damage critically involves neutrophil extravasation from the vasculature into the affected tissue [21]. As our in vitro data showed that SUMOylation can affect the ability of neutrophil migration, we decided to further examine this aspect in vivo using TAK-981. Specifically, we examined neutrophil migration under conditions that minimize the influence of the tissue-specific factors, and studied neutrophil migration into an inert, subcutaneously implanted polyvinyl sponge. As outlined in Figure 6, we found that systemic inhibition of SUMOylation with TAK-981 significantly reduced the numbers of neutrophils that infiltrated the sponge (2.8 ± 0.4 × 10^5^ neutrophils in vehicle control vs. 1.6 ± 0.34 × 10^5^ in TAK treated animals). Together, our results strongly suggest that neutrophil infiltration into inflammatory target tissues is modulated by SUMOylation.

## 3. Discussion

Substantial research in recent years has highlighted the important phenotypic and functional plasticity of neutrophils [22,23,24,25]. Our studies originate from the observations that factors prevalent at sites of inflammation such as hypoxia, acidosis, or changes in temperature can modulate neutrophil survival and chemotactic properties [26,27,28,29], while at the same time these conditions are known to robustly induce SUMOylation [6]. Thus, we postulated that SUMOylation may lead to neutrophil functional reprogramming. Here, our data demonstrate that factors of the inflammatory microenvironment indeed increase SUMOylation in neutrophils and that blocking SUMOylation significantly alters key neutrophil factor functions, including their migratory and phagocytic abilities. These findings add novel insights into the complex interplay of neutrophils with the inflamed tissue and identify SUMOylation as an important mechanism in neutrophil tissue reprogramming.

SUMOylation has emerged as a critical link between the sensing of environmental stress and resultant, rapid changes in the function, stability or intracellular location of key protein regulators of cellular stress responses [11,30,31]. However, as many pathways appear to be SUMOylated at multiple sites, research on specific SUMO target proteins has yielded conflicting results on how this post-translational modification impacts net cellular behavior. Consequently, various groups including our own have focused on global SUMOylation patterns to understand how changes in these patterns effectively reprogram cellular stress responses [12,32,33,34]. In our current work, we demonstrate that the levels of SUMO2/3 conjugation rapidly increased in neutrophils when they were exposed to environmental or inflammatory stimuli. These findings were reproducible using different stimuli (hypothermia, oxidative stress, LPS, and PMA) and were evident in isolated neutrophils from both mice and human volunteers. In addition, we observed a significant increase in leukocyte SUMOylation in a complex clinical setting (hypothermic circulatory arrest surgery of the heart) that combines changes in temperature and robust inflammatory activation. The fact that SUMO conjugation levels rapidly increase with a given stimulus and also rapidly decrease once homeostatic conditions are reinstated, strongly suggests that SUMOylation is an important and highly dynamic switch, regulating neutrophil responses to environmental conditions.

To study how SUMOylation impacts neutrophil functions, we based our experimental strategy on the finding that the various environmental and inflammatory stimuli employed in our study caused a marked increase in SUMOylation and therefore aimed to examine the effect of blocking this response. We found that TAK-981 effectively blocked the global SUMOylation response and, consistent with published reports [16,35,36], had no toxic effects in vitro or in vivo. TAK-981 functions by forming a SUMO–TAK-981 adduct within the catalytic site of the SUMO-activating enzyme (SAE) [16], which prevents the transfer of SUMO isoforms to the E2 conjugating enzyme Ubc9 and consequently to target proteins. While this reaction is highly specific, it is a limitation of our study that off-target effects of the drug cannot be fully excluded. However, this pharmacologic approach provides an opportunity to specifically inhibit the SUMOylation process while avoiding problems previously reported with genetic knock-out strategies. For example, the global deletion of SUMO2 causes embryonic lethality [37], while the knock-out of SUMO1 leads to the compensatory overexpression of SUMO2/3 [32,38]. In our assays, we focused on SUMO2/3 as the isoforms undergoing the most pronounced changes under conditions of stress and thus as those more prominently involved in the ensuing dynamic responses. Because TAK-981 also decreases global SUMO1 conjugation levels [39], our work thus does not allow conclusions on the specific contribution of different SUMO isoforms.

Notably, our studies using TAK-981 in isolated primary murine neutrophils reveal that blocking SUMOylation significantly reduced neutrophil migration toward a fMLP gradient and blunted neutrophil phagocytosis. However, TAK-981 did not appear to affect PMA-stimulated NETosis, indicating that SUMOylation has differential effects on neutrophil functions. It has become evident that interactions with endothelial cells, but also the exposure to the tissue and inflammation specific environment during transendothelial migration, trigger important changes in neutrophil effector cell functions [3,40]. Consequently, our data suggest that the environmental conditions present at sites of inflammation increase neutrophil SUMOylation, which plays a key role in the peripheral reprogramming of neutrophils at inflammation sites.

While SUMOylation has been shown to be an important regulatory mechanism in innate immunity [12,13], its specific role in neutrophil function has been limited to linking SUMO protease 5 (SENP5) expression to the neutrophil differentiation of acute myeloid leukemia (AML) cells [41]. Consequently, we aimed to establish the in vivo relevance of our findings from isolated murine neutrophils. Here, a limitation of our pharmacological approach with TAK-981 is that its administration to animals does not allow specific targeting of neutrophils and therefore its effects on other cell types cannot be ruled out. For example, in our previous work, we had shown that epithelial SUMOylation following intestinal I/R injury is an inflammation-limiting process that reduces epithelial chemotactic signaling and consequently neutrophil infiltration [32]. However, to limit potential effects of TAK-981 on target tissue responses, we employed a sterile sponge model and found that consistent with our in vitro findings, blocking SUMOylation in vivo significantly reduced neutrophil infiltration into the sponge. Thus, our data suggest that blocking global SUMOylation with TAK-981 significantly reduced the neutrophil’s ability to transmigrate into target tissues.

It is noteworthy that our findings on neutrophil migration appear in the context of a large body of work in cancer and wound healing, clearly supporting an important role of SUMOylation in regulating various pathways involved in cellular motility and cell adhesions. For example, SUMOylation appears to modulate the focal adhesion pathway either by directly modifying the key component talin [42], or via SUMOylation of PY2K and the resultant enhanced phosphorylation of downstream components, such as paxillin [43]. On the other hand, SUMOylation of the intermediate filament protein vimentin is important for its dynamic disassembly and blocking such SUMOylation markedly reduced migration of HEK293 cells [44]. Together, this has made SUMOylation a highly interesting target in adjunctive cancer therapy leading to early-phase clinical trials using TAK-981 [45].

Neutrophil transmigration/extravasation across the endothelial cell barrier constitutes a multistep program mediated by a sequence of intricate interactions between leukocytes and the endothelial apical surface and is driven by a set of chemotactic gradients created by cytokines, lipid mediators, bacterial peptides, and peptides from damaged cells [40]. As a consequence, it is by nature a process characterized by extensive crosstalk between neutrophils and their inflammatory microenvironment. Our finding that SUMOylation is a key modifier here offers an intriguing new perspective for the understanding of how neutrophil functions are adjusted as they transmigrate. In addition, the fact that this process can be blocked with TAK-981 opens the possibility to therapeutically limit the role of neutrophil in inflammation-associated damage by modulating its migration into the tissue.

## 4. Materials and Methods

### 4.1. Human Samples

All studies involving volunteers or patients were approved by Duke University IRB, and samples were drawn only after obtaining informed consent. Neutrophils from volunteers were harvested by venipuncture of 6 mL of blood into acid citrate dextrose (ACD) containing buffer and isolation of neutrophils using Polymorphprep (Serumwerk Bernburg, Bernburg, Germany) following the manufacturer’s instructions. After harvesting the polymorphonuclear leukocyte band, the remaining red blood cells were lysed using hypotonic lysis. Neutrophils were then washed in HBSS buffer without Ca^2+^/Mg^2+^ and manually counted in a hemocytometer. Preparations used for further experiments contained >96% live cells as established by trypan blue exclusion. Before conducting experiments, cells were resuspended at a concentration of 5 × 10^6^ cells/mL in RPMI 1640 with 11 mM HEPES pH 7.2 and rested for 1 h at 37 °C.

Patient blood samples were obtained as part of a prospective multi-center clinical trial (https://www.clinicaltrials.gov; NCT02834065) investigating the effects of varying degrees of cooling in randomized patients aged 18 years or older undergoing elective aortic arch surgery (hemi- or total arch) via median sternotomy with hypothermic circulatory arrest and antegrade cerebral perfusion [46]. Exclusion criteria were as per the parent study and were targeted to the primary outcome variables examining neurologic outcomes. Samples for this study were obtained exclusively at the Duke site and were drawn at the baseline (Timepoint 1: after induction of anesthesia but before incision), after initiation of cardiopulmonary bypass and cooling of the body to a target temperature below 34 °C (Timepoint 2), 10 min after completion of circulatory arrest (Timepoint 3), and 10 min after rewarming and separation from cardiopulmonary bypass (Timepoint 4). A total of 9 mL of whole blood was mixed immediately upon harvest with 1 mL ACD buffer and transferred on ice. To prevent changes in SUMOylation levels ex vivo, samples were maintained at 4 °C throughout and each solution used during the isolation procedure was admixed with 20 mM of the SUMO protease inhibitor N-ethylmaleimide (Sigma-Aldrich, #04260, St. Louis, MO, USA) [47]. Blood samples were immediately spun for 10 min at 350× *g* to remove platelet rich plasma before lysis of red blood cells using hypotonic lysis. Leukocytes were then isolated by centrifugation. After washing in PBS, the remaining cell pellet was lysed in Western blot lysis buffer containing phenylmethylsulfonyl fluoride (PMSF) and protease inhibitor as described previously, sonicated, and heat inactivated by boiling for 10 min before storage.

Plasma samples were collected by further centrifugation of the platelet-rich plasma which was obtained during leukocyte isolation. After storage at −80 °C, samples were thawed on ice and centrifuged to remove precipitates. Neutrophil elastase was determined by ELISA (R&D Systems, #DY9167-05, Minneapolis, MN, USA) according to the manufacturer’s instructions.

### 4.2. Animals

All procedures involving animals were approved by the Duke University Medical Center Animal Care and Use Committee and were conducted in accordance with the United States Public Health Service’s Policy on Humane Care and Use of Laboratory Animals. C57Bl/6J male mice (3–5 months old) were obtained from the Jackson Laboratory (Bar Harbor, ME, USA). All animals were housed in a 14:10 light:dark cycle, with ad libitum access to food and water. Animals were assigned randomly to experimental groups, with group assignments blinded until data analysis.

### 4.3. Neutrophil Isolation from Mouse Bone Marrow

An immunomagnetic selection method was used to obtain highly purified neutrophils from mouse bone marrow [48]. Briefly, femoral and tibial bones were collected and cleaned. Bone marrow was harvested using a centrifugation-based method as described previously [49]. Red blood cells were lysed with RBC lysis buffer (BioLegend, #420302, San Diego, CA, USA) for 1 min at room temperature. After washing with PBS, bone marrow cells were filtered with a 70 μm cell strainer, followed by centrifugation for 10 min at 300× *g* at 4 °C. Cells were then resuspended in 200 μL of MACS buffer. Neutrophil purification was then carried out according to the manufacturer’s protocol (Miltenyi Biotec, Neutrophil Isolation Kit mouse, #130-097-658, Bergisch Gladbach, Germany). Purified neutrophils were counted and used for downstream assays.

### 4.4. Western Blotting

Cell pellets were lysed using lysis buffer supplemented with 2% SDS, followed by brief sonication. After centrifugation, the supernatants were collected as protein samples. A standard Western blotting protocol was followed. Signal intensities were quantified using ImageJ2 V2.16.0 (NIH, Bethesda, MD, USA). Two primary antibodies used were anti-SUMO2/3 (Covance, Princeton, NJ, USA) and anti-β-actin (Sigma-Aldrich, #A3854).

### 4.5. NETosis Assay

Freshly isolated neutrophils were resuspended at a concentration of 1 × 10^6^ cells/mL in 1 mL of RPMI1640 medium containing 0.5% BSA. Cells were pretreated with TAK-981 (5 μM; Cayman, #32741, Ann Arbor, MI, USA) or vehicle for 1 h at 37 °C with 5% CO_2_. Then, cells were stimulated with phorbol-12-myristate-13-acetate (PMA; 50 nM) and at the same time, 25 μM SYTOX Green (ThermoFisher, #S7020, Waltham, MA, USA) was added to stain extracellular DNA. After incubating for 2 h, NETosis formation was assessed by measuring the percentage of SYTOX Green-positive cells in each well using flow cytometry [48].

### 4.6. Phagocytosis Assay

Phagocytosis was assessed using pHrodo Red zymosan bioparticles (ThermoFisher; #P35364). Purified neutrophils were seeded at a density of 1 × 10^6^ cells per well in 200 μL of culture medium (10% FBS and 1%PS in RPMI 1640). Cells were pretreated with TAK-981 (5 μM) or DMSO (vehicle) for 1 h, followed by the addition of zymosan bioparticles (40 μg/mL). After a 1-h incubation, cells were washed with PBS and resuspended in FACS buffer (0.5% BSA and 2 mM EDTA in PBS) for flow cytometry analysis. Of note, the pH-sensitive pHrodo Red zymosan bioparticles emit fluorescence only upon internalization.

### 4.7. Transwell Migration Assay

To assess neutrophil migration in response to chemotactic signals, a transwell system was used (VWR, #10769-240, Allentown, PA, USA). A cell suspension (5 × 10^5^ neutrophils/well) in 300 μL RPMI1640 containing 10% FBS was placed in the upper chamber. Cells were pretreated with TAK-981 (5 μM) or DMSO (vehicle) for 1 h. Next, 700 μL of RPMI1640 containing the chemoattractant *N*-formylmethionyl-leucyl-phenylalanine (fMLP; 10 μM) was added to the lower chamber. After a 2-h incubation, cells that had migrated through the transwell membrane into the lower chamber were collected and resuspended in FACS buffer. Cells were then stained with anti-Ly6G antibody for accurate neutrophil counting by flow cytometry.

### 4.8. Subcutaneous Polyvinyl Alcohol (PVA) Sponge Implantation

Sterile PVA discs (10 mm diameter) were used as the implanted material. Mice were anesthetized with isoflurane, and a midline dorsal incision was made. A PVA disc was aseptically implanted into a subcutaneous pocket, and the skin was closed with sutures. Immediately following the procedure, mice received an intraperitoneal injection of TAK-981 (7.5 mg/kg) or corn oil (vehicle), with treatments randomized and mixed within cages [50]. After 24 h, the sponge discs were removed under sterile conditions and placed in a 24-well plate containing 1 mL of pre-chilled PBS per well. Cells were recovered by gently squeezing the sponge 30 times with a 1 mL syringe plunger, with this process repeated four times in fresh wells. The combined cell suspensions from all four wells were centrifuged for 10 min at 300× *g*. Cell pellets were resuspended in FACS buffer and stained for flow cytometry analysis.

### 4.9. Flow Cytometry

Flow cytometry analysis was performed essentially as described [49]. A total of 80 μL of cells were loaded into each well of a 96-well V-bottom plate and incubated with 1 μL of Fc receptor blocker (BioLegend) at 4 °C for 15 min. Then, a mixed antibody cocktail (19 μL) was added to each sample, followed with 30-min incubation at 4 °C in the dark. After washing, cells were resuspended in 100 μL PBS with LIVE/DEAD Blue stain (ThermoFisher) and incubated for 15 min. After washing, cells resuspended in FACS buffer and analyzed on an Aurora flow cytometer (Cytek, Fremont, CA, USA).

### 4.10. Statistical Analysis

All analyses were performed using GraphPad Prism 10 software. Data are shown as mean ± SEM. For comparison between two groups, the unpaired Student’s *t*-test was used. For comparisons involving more than two groups, one-way ANOVA with post hoc Holm–Sidak correction for multiple comparisons was applied. Statistical significance was set as *p* < 0.05.

## Data Availability

Data presented in this study is contained within the article and Supplementary Materials. Further inquiries can be directed to the corresponding author.

## Supplementary Materials

The following supporting information can be downloaded at: https://www.mdpi.com/article/10.3390/ph18071070/s1, Figure S1: Western blot analysis of SUMOylation in patient samples (Supplemental to Figure 1).
