# Supplementary material for: SUMOylation Regulates Neutrophil Phagocytosis and Migration
Source: Pharmaceuticals (Basel). 2025 Jul 20;18(7):1070. doi: 10.3390/ph18071070 (PMC12298196; doi:10.3390/ph18071070)
Supplement: Supplementary file 1 [file pharmaceuticals-18-01070-s001.zip › pharmaceuticals-3706146-supplementary.pdf]

Supplemental data

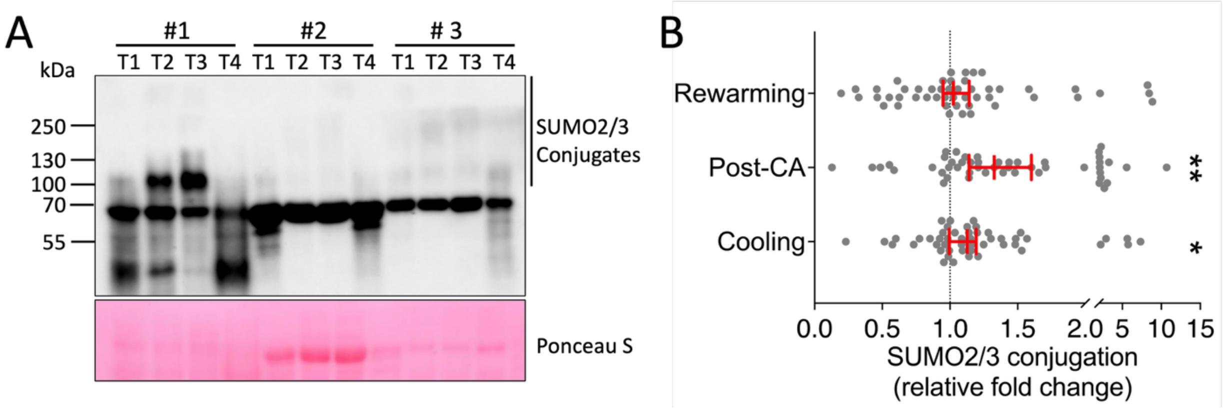

Figure S1. Western blot analysis of SUMOylation in patient samples (Supplemental to Fig. 1). A) Representative Western blot showing samples from 3 individual patients. **B**) Quantification of SUMOylation levels in leukocytes enriched from blood samples of 49 patients. Data are presented as median values with 95% confidence intervals; each dot represents an individual sample. *, $p < 0.05$; **, $p < 0.01$.
